# Gut microecology: effective targets for natural products to modulate uric acid metabolism

**DOI:** 10.3389/fphar.2024.1446776

**Published:** 2024-08-28

**Authors:** Hui Wang, Yixuan Zheng, Mengfan Yang, Lu Wang, Yao Xu, Siqi You, Nan Mao, Junming Fan, Sichong Ren

**Affiliations:** ^1^ Chengdu University of Traditional Chinese Medicine, Chengdu, China; ^2^ Chengdu Medical College, Chengdu, China; ^3^ Department of Nephrology, First Affiliated Hospital of Chengdu Medical College, Chengdu, China; ^4^ TCM Preventative Treatment Research Center of Chengdu Medical College, Chengdu, China

**Keywords:** natural products, gut microecology, gut flora, gut barrier, uric acid, hyperuricemia

## Abstract

Gut microecology,the complex community consisting of microorganisms and their microenvironments in the gastrointestinal tract, plays a vital role in maintaining overall health and regulating various physiological and pathological processes. Recent studies have highlighted the significant impact of gut microecology on the regulation of uric acid metabolism. Natural products, including monomers, extracts, and traditional Chinese medicine formulations derived from natural sources such as plants, animals, and microorganisms, have also been investigated for their potential role in modulating uric acid metabolism. According to research, The stability of gut microecology is a crucial link for natural products to maintain healthy uric acid metabolism and reduce hyperuricemia-related diseases. Herein, we review the recent advanced evidence revealing the bidirectional regulation between gut microecology and uric acid metabolism. And separately summarize the key evidence of natural extracts and herbal formulations in regulating both aspects. In addition,we elucidated the important mechanisms of natural products in regulating uric acid metabolism and secondary diseases through gut microecology, especially by modulating the composition of gut microbiota, gut mucosal barrier, inflammatory response, purine catalyzation, and associated transporters. This review may offer a novel insight into uric acid and its associated disorders management and highlight a perspective for exploring its potential therapeutic drugs from natural products.

## 1 Introduction

In recent years, there has been plenty of research interest in the role of gut microecology in regulating various physiological and pathological processes (Shen et al., 2023). The human gut microbiota, composed of trillions of microorganisms residing in the gastrointestinal tract, is crucial in maintaining overall health and metabolism (Li et al., 2024), and recent studies indicated that it is closely involved in regulating uric acid (UA) metabolism (Dang et al., 2023). For instance, certain gut microorganisms play a beneficial role in UA metabolism, facilitating the breakdown of purine and promoting the excretion of UA through the intestinal tract (Wang J. et al., 2022; Kasahara et al., 2023). On the contrary, many investigations implicated that an altered gut microecology could produce enzymes that contribute to the conversion of purine into UA or impair the excretion of UA, resulting in its accumulation and the development of hyperuricemia (HUA) (Wang Z. et al., 2022; Yin et al., 2022). These inconsistent data indicated the bidirectional effect of gut macroecology on UA metabolism, implying that a favorable interaction between gut microecological homeostasis and UA metabolism balance may be a candidate biomarker for health monitoring and drug target screening.

Natural products, including monomers, extracts, and herbal formulations derived from various sources such as plants, animals, and microorganisms, have been shown as potential drugs to modulate gut microecology and UA metabolism for body health (Guo et al., 2017; Yang L. et al., 2022). It testified that some plant extracts contain bioactive compounds such as polyphenols (Ye et al., 2022), flavonoids (Oteiza et al., 2018), which can selectively promote beneficial bacteria growth while inhibiting harmful ones. In addition, Chinese medicine herbal formulation compounds such as puerarin, glycyrrhizin, berberine, and baicalin significantly repair the gut barrier and reduce pro-inflammatory cytokine expression in the gastrointestinal tract (Wu et al., 2019). Besides regulating gut macroecology, certain extracts and herbal formulations possess UA-lowering properties, which can inhibit the production via diminishing xanthine oxidase (XO) activity or enhance the excretion of UA by adjusting UA transporters, thereby maintaining its physiological levels in the body (Xu et al., 2024). This viewpoint of natural products targeting gut microecology to modulate UA metabolism has recently been accepted theoretically. Considering the interwinding effect on gut microbiota and UA interactively, researchers reasoned that natural products could adjust UA balance via modulating gut microbiota composition, typically engaging an augmentation of beneficial bacteria and a diminution of harmful bacteria (Wang Z. et al., 2022). In recent years, many advanced studies have testified that natural products not only modulate gastrointestinal flora composition but also enhance gut barrier function, attenuate inflammatory responses, modulate purine metabolism, and regulate transporter activity (Bian et al., 2020; Mehmood et al., 2024). In summary, natural products showed promising potential for modulating the interwinding effect between gut microecology and UA metabolism. Therefore, we review the bidirectional regulating relationship between gut microbiota and UA metabolism and summarize natural products targeting gut microecology to modulate UA metabolism. It may provide an innovative avenue for developing natural products interventions that can effectively modulate UA metabolism and mitigate the risk of associated diseases.

## 2 Overview of uric acid metabolism and its functions

**FIGURE 1:**
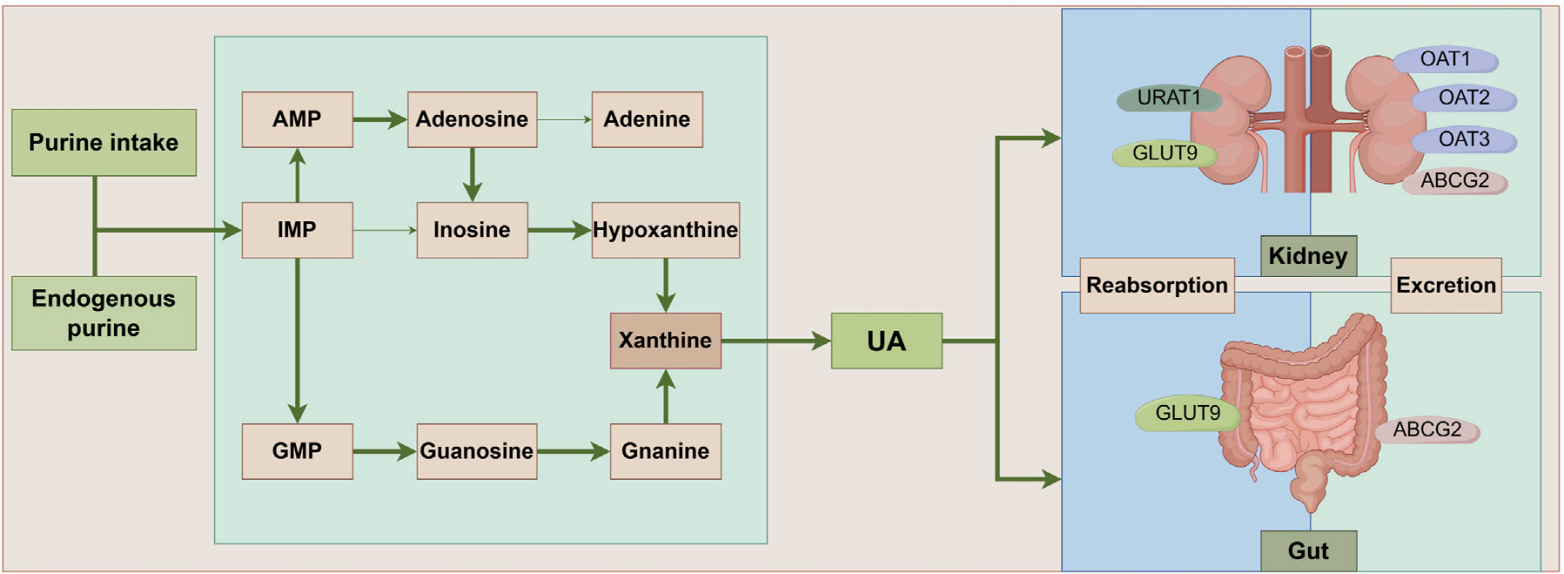
UA metabolic pathways. Ingested and endogenously produced purines impose a significant strain on the body’s purine nucleotide synthesis processes, culminating in the accumulation of intermediates such as IMP, AMP, and GMP. This accumulation triggers an elevated conversion of xanthine, facilitated by xanthine oxidase, ultimately leading to the generation of UA. Notably, approximately one-third of UA undergoes metabolism within the intestinal tract, with UA-associated transporter proteins playing pivotal roles in both the reabsorption and excretion of UA. ABCG2: ATP-Binding Cassette Subfamily G Member 2; AMP: Adenosine Monophosphate; GLUT9: Glucose Transporter 9; GMP: Guanosine Monophosphate; IMP:Inosine Monophosphate; OAT1: Organic Anion Transporter 1; OAT2: Organic Anion Transporter 2; OAT3: Organic Anion Transporter 3; UA: uric acid; URAT1: Uric Acid Transporter 1.

UA is a nitrogenous product resulting from the breakdown of purine nucleotides. It plays a significant role in the human body, acting as an antioxidant and participating in various physiological and pathological processes. The liver, gut, kidneys, and vascular endothelium are the primary tissues for UA metabolism. Endogenous UA production commences with the catabolism of nucleic acids, followed by conversion to xanthine via a series of enzymes, ultimately yielding UA through the action of XO (El Ridi and Tallima, 2017). Differently, exogenous UA synthesis is primarily derived from ingesting of purine-rich foods. Approximately one-third of UA excretion occurs in the gastrointestinal tract, with the remainder excreted from urine in the kidney via transporters (Yin et al., 2022) (Figure 1). Studies demonstrated that UA had a bidirectional role in regulating the body’s health, and a U-shaped correlation has been established between UA levels and total mortality (Crawley et al., 2022). A literature displayed that UA is a potent antioxidant, rivaling the antioxidant capacity of ascorbic acid, and possesses multifaceted functions, including blood pressure maintenance, anti-aging, and neuroprotection (Wen et al., 2024). However, elevated UA levels of HUA are associated with a spectrum of diseases, such as gout, kidney stones, chronic renal disease, hypertension, and metabolic syndrome (So and Thorens, 2010; Alvarez-Lario and Macarron-Vicente, 2011; Yanai et al., 2021). HUA is defined as blood UA levels exceeding 7.0 mg/dL for males or 5.7 mg/dL for females due to UA production surpassing its elimination (Yang B. et al., 2022). Of note, young males currently exhibit an increasing prevalence of HUA, and its incidence rate among Chinese adults was up to 24.4% (Zhang et al., 2021b). Thus, we should pay serious attention to UA metabolism and its homeostasis modulation for human health.

## 3 Gut microecology and uric acid metabolism

### 3.1 Mutual favorable effect between gut microbiota and uric acid metabolism

**FIGURE 2:**
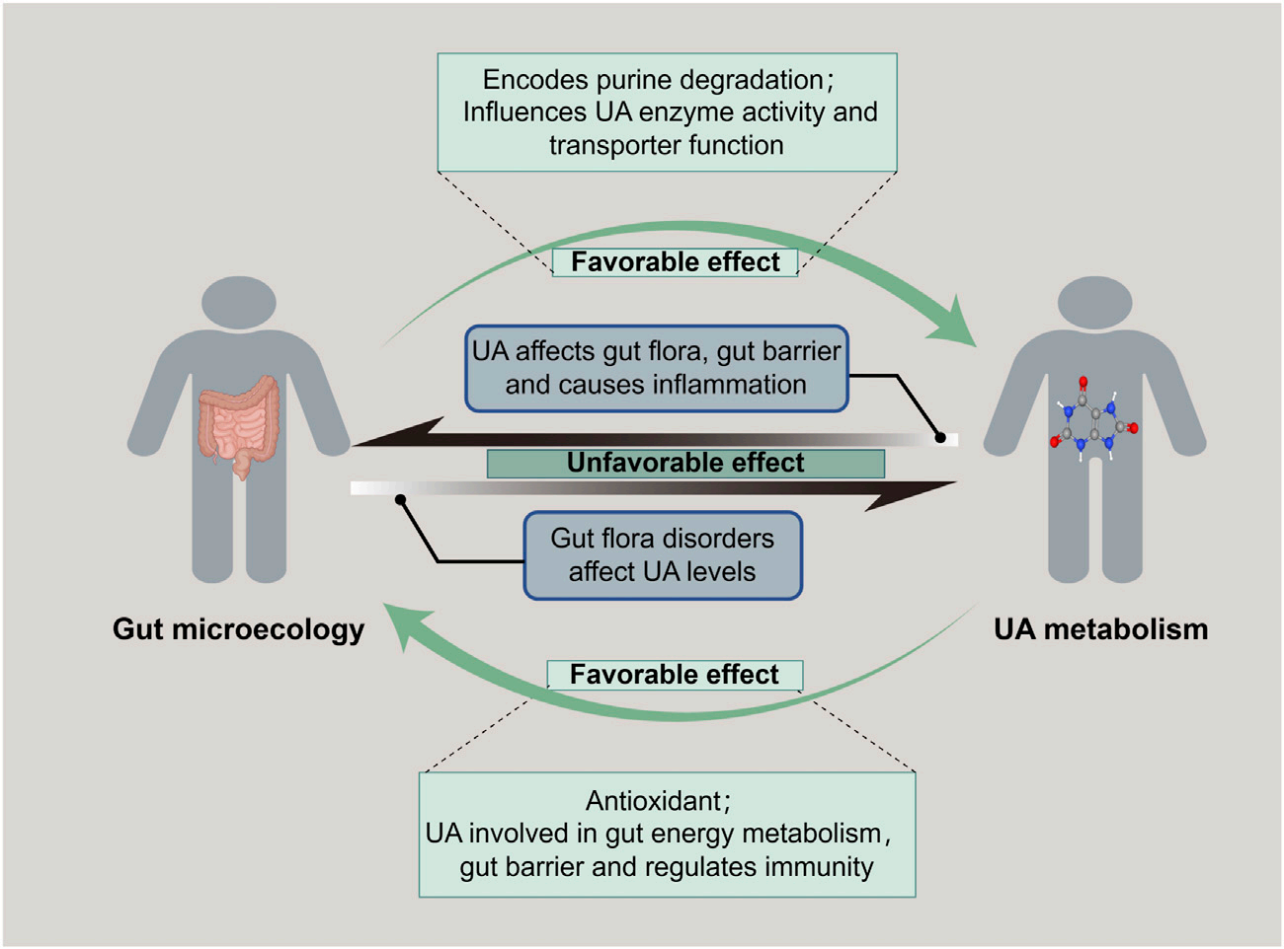
Bidirectional regulation of gut microecology and UA metabolism. Gut microecology promotes both purine degradation to reduce UA level and UA synthesis to increase UA level. While UA is related to gut energy metabolism and maintenance of gut barrier, it also affects the gut flora, destroys the mucosal barrier, and causes an imbalance of gut microecology.

The gastrointestinal tract harbors a diverse ecosystem of microorganisms known as the gut microbiota. These microorganisms maintain a symbiotic relationship with the human host and play a vital role in various aspects of health, including immune regulation, nutrient absorption, and metabolism. Emerging evidence suggests that the gut microbiota significantly influences UA metabolism (Wang J. et al., 2022). To maintain the homeostasis of UA, the gut bacteria adapt to the host’s microenvironment, serving as a crucial regulator of UA production, metabolism, and excretion pathways. The purine degradation clusters have been identified in diverse gut bacterial groups, including *Bacillota*, *Fusobacteriota* and *Pseudomonadota* (Kasahara et al., 2023). Besides, changes in metabolizing gene clusters facilitate UA’s metabolic conversion into hypoxanthine or short-chain fatty acids (SCFAs) (Liu et al., 2023b). Hypoxanthine plays a pivotal role in energy metabolism and safeguards the gut barrier integrity mediated by gut epithelial cells (Lee et al., 2018). SCFAs are involved in gut endocrine and immune modulation, contributing to the treatment and recovery of various metabolic diseases (van der Hee and Wells, 2021). In addition, an investigation implied that an increase in the abundance of beneficial gut bacteria correlates with the upregulation of UA metabolism-related excretory genes, such as ATP-binding cassette sub-family G member 2 (ABCG2) and Organic Anion Transporter 1 (OAT1), and the downregulation of absorption genes, including Urate Transporter 1 (URAT1) and Glucose Transporter 9 (GLUT9) (Li et al., 2021b). For instance, *Lactobacillus*, a member of the gut flora, exhibits inhibitory effects on the XO and purine nucleoside phosphorylase while promoting the activity of nucleoside hydrolase RihA-C to curtail urate synthesis, and it upregulates ABCG2, enhancing UA excretion as well (Li M. et al., 2023). These studies indicated that the gut microbiota and its metabolites regulate gut microecological functions and facilitate normal UA metabolism (Figure 2).

Conversely, UA plays a pivotal role in reverse-modulating the microbiota of the gastrointestinal tract, in addition to the usual consideration of safeguarding cardiac, vascular, and neuronal cells from oxidative stress as a physiological antioxidant. A report evidenced that appropriate levels of UA have conferred an evolutionary advantage on humans by elevating antioxidant markers and fostering the diversity of gut flora (Wada et al., 2022). Moreover, UA is indispensable for free radical scavenging and offers a fundamental tissue repair mechanism that benefits gut microbiota (El Ridi and Tallima, 2017). Purine, a source of carbon and energy for gut bacteria, is well-known as the precursor of UA synthesis, which is also frequently implicated in the auto-regulation of gut microecology (Kasahara et al., 2023). Notably, in healthy individuals with normal levels of UA, butyrate-producing bacteria are more abundant, and other beneficial bacteria, such as *Bifidobacterium* and *Clostridium sensustricto1*, are significantly enriched. In turn, these dominant microflora collectively maintain the integrity of the gut barrier, exert immunomodulatory effects, and possess anti-inflammatory properties for favoring UA metabolism (Mendez-Salazar et al., 2021). It has indicated that an appropriate level of UA is crucial for maintaining gut ecological flora diversity to modulate metabolic homeostasis favorably.

### 3.2 Reciprocal unfavorable effect between dysregulated gut microbiota and uric acid metabolism

A diminution of beneficial bacteria and a proliferation of harmful bacteria within the gut microbiota can incite microecological imbalance, which perturbs UA metabolism, consequently enhancing UA production and diminishing excretion, ultimately resulting in HUA. A Mendelian randomization study has implicated thirty distinct gut microbiota species in modulating UA levels, with five specific types exhibiting a notable influence on blood UA concentrations (Wang et al., 2023). Various gut bacteria exert diverse effects on UA metabolism. Studies have shown that elevated *yeast* proliferation and decreased *bifidobacteria* abundance facilitate purine metabolism and augment UA production (Chiaro et al., 2017; Gong et al., 2022). Furthermore, the disruption of gut microbiota induced by various causal agents can also significantly impact UA levels. Recent literature demonstrated that antibiotic-induced alteration in gut microbiota resembled that observed in animals with HUA, with a reduced abundance of purine salvage proteins expression in the gut microbiota, increasing the risk of HUA (Liu et al., 2023). In addition, exposure to the heavy metal nickel suppressed beneficial gut bacteria such as *Lactobacillus* and Lachnospiraceae, while promoting the proliferation of harmful bacteria like *Parabacteriodes* and *Escherichia-Shigella* (Yang et al., 2023). This impairment in purine degradation elevates UA levels and systemic inflammatory response. In this case, gout can be triggered by the disruption of the gut barrier and the subsequent release of inflammatory factors, stemming from a decrease in SCFAs, a metabolite of the gut microbiota, and an increase in lipopolysaccharides (Liu et al., 2022). These indicated that disorder modulation of UA resulting from an imbalance of gut microecology is a common cause for various diseases.

On the contrary, elevated levels of UA have a significant effect on gut microecology reciprocally. A study unveiled that distinctive miRNAs associated with the onset and remission of UA in *Apostichopus japonicus* hydrolysate. These miRNAs exhibited a strong correlation with the metabolism of tryptophan, bile acid, and SCFAs, suggesting a bidirectional interplay between UA metabolism, gut bacteria and their metabolites (Fan et al., 2022). A further discovery of distinct gut bacterial flora in patients with varying UA levels. In particular, the abundance of enterobacteria capable of producing SCFAs, such as *Ruminococcus*, was decreased, while the abundance of *Proteobacteria* and *Bacteroides* was increased in patients with HUA (Liang et al., 2022). These findings support the hypothesis that gut flora and microbial markers could be predictive models for HUA. Studies have shown that with escalated UA levels, the bacterial flora diversity was diminished, which led to a downregulation of tight junction proteins Occludin and Claudin-1, compromising the gut barrier, enhancing permeability and promoting an imbalance between Th17 and Treg cells in the gut (Lv et al., 2020; Wang P. et al., 2022). The downregulated of UA-secreted transporter proteins ABCG2, OAT1, and OAT3, while upregulated of the UA reabsorption transporter protein URAT1, which contributed to HUA and its continuous secondary disadvantage of the gut microecology (Wu et al., 2022). Notably, cultivating beneficial bacteria, such as *lactobacilli*, can produce SCFAs and mitigate the severity of HUA and its associated secondary damage (Wan et al., 2020). In a word, abnormally elevated UA can disrupt gut flora’s composition, compromise the gut barrier, and subsequently trigger imbalanced gut microecology modulation.

## 4 Natural products are innovative modulators of gut microecology

Natural products are compounds derived from natural sources, such as plants, animals, or microorganisms, encompassing a plenty of substances, including monomers, extracts, and herb formulations. In recent years, many investigations have revealed that natural products were innovative drugs with the potential to modulate gut microecology and subsequently regulate UA metabolism. Because more research is interested in the role of extracts and herb formulations modulating the gut microbiota balance, thus we enumerate their action below.

### 4.1 Natural extracts engage in modulating gut microecology

The advent of liquid chromatography-mass spectrometry (LC-MS) has ushered in a new era of precision in the effective employment of natural products, enabling the accurate determination of phytochemical extract profiles and predictive assessment of plasma absorption capacity based on the molecular weight and lipophilicity of individual chemical components (Selby-Pham et al., 2018). Under such conditions, numerous natural products, particularly these extracts in managing gut microecology, have garnered extensive attention. It has been evidenced that extracts such as *Rhodiola crenulata* extract (Wang et al., 2021), *Ginkgo biloba* leaves extract (Wang Y. et al., 2022), *Centella asiatica* ethanol extract (Li H. et al., 2021), have exhibited promising potential in alleviating gut inflammation by reparative actions on the gut mucosal barrier and modulation of gut flora composition. It was noteworthy that individual natural product preparations exhibited significant therapeutic value. For instance, the Wuzhi capsule, derived from *Schisandra chinensis* extract, is a clinically recognized synergistic and detoxifying formulation, that can mitigate gut microecological dysbiosis induced by mycophenolate through the inhibition of oxidative stress, inflammation, and apoptosis (Zhang et al., 2022c). In the realm of natural products, our investigation revealed that polysaccharides and polyphenols emerge as potent material foundations for meticulously managing gut microecology (Lin et al., 2019; Pascuta et al., 2022). Diverse polysaccharide fractions, encompassing those derived from *Lycium barbarum polysaccharides* (Gao et al., 2021), marine algal polysaccharides (Yu et al., 2023), alongside polyphenolic fractions such as blueberry polyphenols (Polewski et al., 2020), tea polyphenols (Ye et al., 2022), exhibit remarkable capability of regulating bacterial flora composition, whereby benefit for bolstering the gut mucosal barrier, mitigating oxidative stress, and gut inflammation. Meanwhile, a nuanced analysis of the intricate relationships between dietary polyphenols and the gut microbiota further verified that polyphenols can modulate gut microecology (Koudoufio et al., 2020). Interestingly, many innovative natural antimicrobials have been developed with the rapid progress in drug discovery based on natural extracts modulating gut microecology in recent years (Guglielmi et al., 2020). It represents a cornerstone of screening natural products for combating human health threats of gut microbiota-related disease and paves a new avenue for natural products to be used in clinical applications.

### 4.2 Herbal formulations and their ingredients based on clinical efficacy modulate gut microecology

Natural products constitute a pivotal source of herbal remedies in traditional Chinese medicine. Herbal formulations contain massive natural ingredients owning multifaceted pharmaceutical targets and diverse medical therapeutic efficacy. Due to their pharmaceutical activity, these formulations are applied in clinical treatment as pharmaceutical prescriptions in China. Studies have testified that many herbal formulations and their active ingredients exhibited a role in sustaining gut microecological stability with an intricate reciprocal complementary mechanism (Zheng et al., 2022). However, pieces of literature implied that ingredients within herbal formulations displayed vast heterogeneity. For instance, in a mouse model of spleen deficiency syndrome, gut barrier impairment, and disrupted microbiota metabolism were observed. It evidenced that the active polysaccharide component S-3 in Sijunzi decoction primarily enhanced gut immune function and gut microbiomes, whereas its non-polysaccharide component was chiefly engaged in ameliorating gut motility disorders (Ma et al., 2021). It evidenced that metabolic pathways of the gut microbiota play a crucial role in regulating gut microecological balance, especially carbohydrates, SCFAs, and amine metabolites, which are associated with the gut flora. A recent study implied that these metabolites are critical targets of Zengye decoction by modulating gut microecology in constipated rats mode (Liu et al., 2019). Besides, another classic prescription Shenling Baizhu San remarkably alleviated gut microecological disorders induced by indigestion, thereby modulating microflora energy metabolism, amino acid metabolism, and other related pathways (Zhang et al., 2020). In conclusion, evidence suggests that the multi-component and multi-target effects feature of herbal formulations is well-suited to modulating the complexity of gut microecology, and this afford them effectively regulate gut microecological homeostasis. Whereas, despite the clinical efficacy of herbal formulations and their ingredients, many researchers still reckon that there will be a long way and substantial challenging work needs to be done for screening feasible clinical natural products drugs (Zhou et al., 2016).

## 5 Natural products regulate uric acid metabolism

Several natural products have shown promising effects in modulating UA metabolism, such as resveratrol and quercetin, have been reported to regulate UA metabolism through the inhibition of XO, a key enzyme in UA production. In general mechanism, studies speculated that natural products mainly switch the activity of key enzymes involved in the production process of UA or target critical transporters of its excretion, thereby orchestrating the balance of UA metabolism.

### 5.1 Natural extracts engage in modulating uric acid metabolism

In recent years, many researchers have manifested that the utilization of natural extracts could effectively regulate the synthesis and excretion metabolism of UA (Zhang X. et al., 2022). XO is a pivotal enzyme for UA production, and its activity can be modulated by administering natural inhibitors (Sun et al., 2024). Compounds of natural extracts such as flavonoids, anthraquinones, and xanthones have been evaluated for their potential inhibitory effect of XO, employing techniques like 3-D QSAR analysis and molecular docking of natural XO inhibitors (Malik et al., 2019). Consistently, flavonoid extracts derived from saffron floral bio-residues have consistently exhibited antagonistic effects against HUA by modifying gut flora associated with host metabolism, inhibiting XO activity, and subsequently reducing UA synthesis (Chen et al., 2022). Intriguingly, in tandem with compound structure-based virtual screening for UA-producing inhibition, isopentenyl dihydro flavones have been identified from a natural herb database, which were as potential activated scaffolds as human urate transporter 1 (hURAT1) inhibitors for the treatment of gout (Chen et al., 2021). These finding emphasized the capability of flavonoids as a primary component for potentially controlling UA metabolism. In addition, tea polyphenols (Zhang G. et al., 2022), apple polyphenols (Cicero et al., 2017), and numerous other polyphenolic compounds also inhibited the activity of XO. Recent studies revealed that many other natural extracts, including the ethanol extract of Amomum villosum Lour (Dong et al., 2023), the active ingredient of Lagotis brachystachya (Zhu et al., 2021) and numerous others, not only inhibited XO but also regulated urate transporter proteins, thereby reducing UA production and promoting excretion. However, the underlying mechanisms of natural extracts involving UA metabolism are rather complicated, which may be influenced by numerous factors, including genetics, gender, health status, etc. Thus, it elicits challenges of natural extracts exerting the modulation of UA metabolism directly. Therefore, screening natural extracts targeting purine degradation, UA transporters, and gut microbiotas or their associated enzymes involved in the UA metabolism process becomes a new highlighting orientation to develop novel therapeutic agents for UA-associated disease treatment (Rullo et al., 2023).

### 5.2 Herbal formulations based on clinical efficacy modulate uric acid metabolism

With the gradually increased prevalence rates of UA dysmetabolism and its associated disorders in recent years, many researchers have oriented to exploring therapeutic natural products for HUA, particularly focusing on herbal formulations in China. Strikingly, it has testified that several herbal formulations and their ingredients exhibited robust clinical efficacy in modulating UA metabolism and mitigating symptoms of UA-related diseases. For instance, classic formulations such as Wuling San (Huang et al., 2023), Siwu decoction (Wang et al., 2016), Fuling-Zexie formula (Lu et al., 2024), and Simiao San (Zhang Y. et al., 2023) have exhibited modulatory effects on UA metabolism, encompassing inhibition of XO activity, modulation of UA transporter protein, regulation of inflammatory signaling pathways, such as NLRP3 complex signaling activity. Additionally, in a clinical trial of an herbal drug treating gouty arthritis, investigators revealed that the ingredients of Huzhang Granule, a traditional Chinese herbal compound, exerted a better capability of analgesic and anti-inflammatory effects, acquired a lower level of UA, even with less incidence of adverse effects, comparing with that of the etoricoxib control group (Wang et al., 2024). Furthermore, it has been reported that the Shuang-Qi gout capsule, a patented pharmaceutical prescription currently utilized in clinical application, exhibited its ability to benefit UA metabolism effectively in a dose-dependent manner, consequently treating gout-related tissue edema and pain (Kodithuwakku et al., 2013). In conclusion, numerous herbal formulations exerted significant efficacy in modulating UA metabolism. However, plenty of challenging work need to be done to clarify the complexity of their ingredient constitution and decipher their underlying mechanisms of UA metabolism modulation.

## 6 Complex mechanism of natural products modulating uric acid metabolism via its targeted gut microecology

**FIGURE 3:**
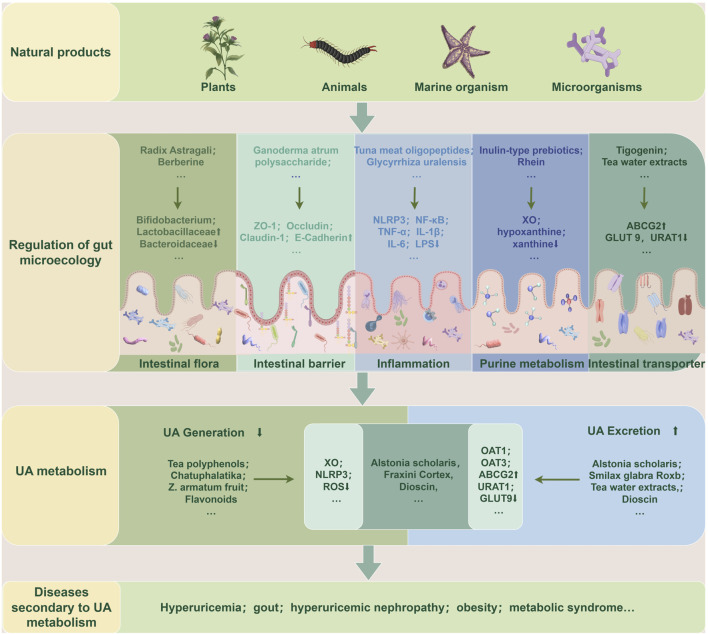
Natural products target gut microecology to regulate UA metabolism and secondary diseases. Extracts or preparations from natural products such as plants, animals, marine organisms, and microorganisms can repair gut microecological imbalance, resulting in reduced UA production or increased excretion, thereby improving UA metabolism and related secondary diseases. The specific ways are to restore the gut flora structure, repair gut barrier function, reduce the inflammatory state, and regulate purine metabolism and transporter activity. ABCG2: ATP-Binding Cassette Subfamily G Member 2; GLUT9: Glucose Transporter 9; IL-1β: Interleukin-1β; IL-6: Interleukin-6; LPS: Lipopolysaccharide; NF-κB: nuclear factor-κB; NLRP3: nucleotide-binding oligomerization domain-like receptor family pyrin domain-containing 3; OAT1: Organic Anion Transporter 1; OAT3: Organic Anion Transporter 3; ROS: Reactive Oxygen Species; TNF-α: Tumor Necrosis Factor-α; UA: uric acid; URAT1: Uric Acid Transporter 1; XO: xanthine oxidase; ZO-1: zonula occludens-1.

As aforementioned, natural products, including various extracts and herbal formulas, can modulate gut microbiota and UA metabolism. It inspired researchers to speculate that an interwinding effect existed between gut microecology and UA metabolism, and natural products could modify gut microecology to orchestrate UA metabolism balance. Recent mechanistic studies indicated that natural products could target gut microbiota to orchestrate an intricate network of modifying its composition, gut mucosal barrier, inflammatory response, purine catalyzation, and associated transporters. Understanding these intriguing mechanisms of these natural products modulating UA metabolism balance may propose new strategies for managing HUA and its associated diseases. (Table 1; Table 2; Figure 3).

**TABLE 1:** Natural extracts target gut microbiome to regulate UA metabolism and antagonize associated disorders.

Mechanism	Natural extracts	Disease models	Outcomes	References
Regulation of gut microbiota composition	Camellia sinensis	PO and adenosine induced HUA mice	Ruminococcus,*Lactobacillus*↑ *Bacteroides*, *Escherichia coli*↓	Wu et al. (2022)
	Coffee leaf tea extracts	High purine diet induced HN rats	*Phascolarctobacterium, Alloprevotella,* and *Butyricicoccus*↑	Zhou et al. (2023)
	Cichorium intybus L. formula	Adenine combined with ethambutol induced HN rats	Lactobacillaceae, Erysipelotrichaceae, Lachnospiraceae, Ruminococcaceae, and *Bifidobacterium*↑ *Bacteroides*↓	Amatjan et al. (2023)
	Chicory	High-purine diet induced HUA quails	*Bifidobacterium,* Erysipelotrichaceae↑ Helicobacteraceae↓	Bian et al. (2020)
	Radix Astragali	PO-induced HUA mice	Lactobacillaceae*,Lactobacillus murine*↑ Prevotellaceae, Rikenellaceae and Bacteroidaceae↓	Deng et al. (2023)
	Berberine	PO-induced HUA mice	*Coprococcus* *,* *Bacteroides* *,* *Akkermansia* and *Prevotella*↑	Shan et al. (2022)
	Kidney tea	PO-induced HUA mice	*Roseburia, Enterorhabdus*↑ *Ileibacterium* and UBA1819↓	Chen et al. (2023a)
	Curcumin	Adenine and PO induced HN rat	*Lactobacillus* and Ruminococcaceae↑ *Escherichia-Shigella* and *Bacteroides*↓	Xu et al. (2021a)
Maintenance of gut barrier’ integrity	Pectic polysaccharides from Aconitum carmichaelii leaves	DSS induced acute ulcerative colitis mice	ZO-1,occludin↑ LPS,NOD1,TLR4 ↓	Fu et al. (2022)
	Marine fish protein peptide	PO induced HUA rats	Recovering the expression of tricellular tight junction protein ILDR2 and the immune-related genes *Ccr7* and *Nr4a3*	Wu et al. (2023)
	Ganoderma atrum polysaccharide	Acrylamide induced intestinal injury in rats	Decreased inflammatory cell infiltration and restoration of intact intestinal epithelium	Yang et al. (2019)
	Extract of Dendrobium officinale leaves	Unhealthy lifestyle induced HUA rats	ZO-1 and occludin↑	Li et al. (2023a)
Modulation of inflammatory response	Apostichopus japonicus oligopeptide	Purine-rich solution induced HUA mice	NLRP3, NF-κB↓ restores m6A methylation levels	Lu et al. (2021)
	Tuna meat oligopeptides	PO combined with purine-rich solution induced HUA mice	NLRP3, TLR4/MyD88/NF-κB↓ the phosphorylation of p65-NF-κB↓	Han et al. (2020)
	Camellia japonica bee polle	PO induced HUA mice	NLRP3↓ TLR4/MyD88/NF-κB↓	Xu et al. (2021b)
	*A. japonicus* (EH-JAP) and A. leucoprocta (EH-LEU)	Diet-induced HUA mouse model	TLR4/MyD88/NF-κB↓ IL-1β, TNF-α, IL-6 ↓ TGF-β, IL-10↑	Wan et al. (2020)
	Glycyrrhiza uralensis	CPT-11 induced colitis mice	TNF-α, IL-1β,IL-6,NLRP3↓ Further regulates UA metabolism	Yue et al. (2021)
Regulation of purine metabolism	Inulin-type prebiotics	Continuous ambulatory peritoneal dialysis patients	Enriched purine-degrading species, enhanced fecal UA degradation	He et al. (2022)
	Rhein	DSS induced mouse colitis model	*Lactobacillus* changed purine metabolism indirectly,led to decreased UA levels	Wu et al. (2020)
	Flavonoid extract of saffron floral bio-residues	PO induced HUA rats	Hepatic XO, UA↓	Chen et al. (2022)
	Enteromorpha prolifera polysaccharide	Hypoxanthine and oteracil potassium induced HUA mice	Serum XO, hepatic XO↓	Li et al. (2021c)
Modulation of gut transporter activity	Stevia residue extract	PO-induced HUA mice	ABCG2↑ GLUT9, UA↓	Mehmood et al. (2019)
	Tigogenin	PO induced HUA rats; adenine-PO induced HUA mice	OAT-1, ABCG2↑ GLUT9, URAT1↓	Zhang et al. (2018)
	MannuronatE oligosaccharide	0.5% Sodium carboxymethyl Cellulose solution containing PO induced HUA mice	Renal GLUT9, URAT1↓ Gut GLUT9↓ Gut ABCG2↑	Wei et al. (2023)
	Tea water extracts	PO and adenosine induced HUA mice	Renal ABCG2, OAT1 and OAT3↑ gut ABCG2↑ Renal URAT1↓	Wu et al. (2022)
	*Ulva lactuca* polysaccharide	Hypoxanthine and oteracil potassium induced HUA mice	ABCG2/OAT1↑ URAT1, GLUT9↓	Li et al. (2021b)

ABCG2: ATP-Binding Cassette Subfamily G Member 2; CPT-11: Irinotecan HCl, Trihydrate; GLUT9: Glucose Transporter 9; HN: hyperuricemic nephropathy; HUA: hyperuricemia; IL-1β: Interleukin-1β; IL-6: Interleukin-6; IL-10: Interleukin-10; LPS: lipopolysaccharide; NLRP3: nucleotide-binding oligomerization domain-like receptor family pyrin domain-containing 3; NOD1: Nucleotide-binding Oligomerization Domain-containing Protein 1; OAT1: Organic Anion Transporter 1; OAT3: Organic Anion Transporter 3; PO:potassium oxonate; TLR4/MyD88/NF-κB:Toll-like receptor 4/myeloid differentiation factor 88/nuclear factor-κB; TGF-β: Transforming Growth Factor-β; TNF-α: Tumor Necrosis Factor-α; UA: uric acid; URAT1: Uric Acid Transporter 1; XO: xanthine oxidase; ZO-1: Zonula occludens-1.

**TABLE 2:** Herbal formulations target gut microbiome to regulate UA metabolism and antagonize associated disorders.

Mechanism	Herbal formulations	Disease models	Outcomes	References
Regulation of gut microbiota composition	Modified Baihu decoction	Sodium urate induced acute gouty arthritis rat	Lachnospiraceae*, Muribaculaceae,* and Bifidobacteriaceae↑ Lactobacillaceae, Erysipelotrichaceae, Ruminococcaceae, Prevotellaceae and Enterobacteriaceae↓	Wang et al. (2022c)
	CoTOL	PO induced HUA rats	Akkermansia↑ *Bacteroides* and Alloprevotella↓	Gao et al. (2020)
	Bi Xie Fen Qing Yin decoction	PO and adenine induced HN mouse model	Ruminococcaceae*, Clostridium sensu stricto 1,* and *Streptococcus*↑ Desulfovibrionaceae*, Enterobacter, Helicobacter,* and *Desulfovibrio*↓	Lin et al. (2024)
	Cichorium intybus L. formula	Adenine combined with ethambutol induced HN rats	Lactobacillaceae, Erysipelotrichaceae, Lachnospiraceae, Ruminococcaceae, and *Bifidobacterium*↑ *Bacteroides*↓	Amatjan et al. (2023)
	Fangyukangsuan granules	PO and hypoxanthine induced HUA rat model	Fermentation of pyruvate to SCFAs↑ amino acid biosynthesis↓	Zhang et al. (2024)
	Guizhi Shaoyao Zhimu Decoction	High-purine diet combined with local injection induced gouty arthritis rat	*Lactobacillus,* Ruminococcaceae, and *Turicibacter*↑ *Blautia*↓	Bian et al. (2024)
Maintenance of gut barrier’ integrity	Qu-zhuo-tong-bi decoction	High-fat diet and MSU crystal-induced gouty arthritis model	SCFAs↑ ZO-1, Occludin↑	Wen et al. (2020)
Modulation of inflammatory response	Sanmiao Wan	Intra-articular injection induced acute gouty arthritis rat	MDR1 mRNA and P-gp↓ knee joint swelling, synovial hyperplasia , inflammatory cell infiltration↓	Wu et al. (2018)
	Si-Miao-San	Monosodium urate induced acute gouty arthritis mice	M2 macrophage polarization↑ PI3K/Akt signaling↓	Cao et al. (2021)
Regulation of purine metabolism	Er Miao Wan	High-fructose diet induced HUA rat	Six purine metabolites related to HUA were changed, including UA, hypoxanthine, xanthine, deoxyadenosine, deoxyguanosine, and deoxyinosine	Gu et al. (2023)
	Qi-Zhu-Xie-Zhuo-Fang	Adenine and PO induced HN rat	XO, renal epithelial-to-mesenchymal transition↓	Huijuan et al. (2017)
Modulation of gut transporter activity	Dendrobium officinalis Six nostrum	PO and hypoxanthine induced HUA rat	ABCG2 and PDZK1↑ Intestinal GLUT9↓	Ge et al. (2023)

ABCG2: ATP-Binding Cassette Subfamily G Member 2; GLUT9: Glucose Transporter 9; HN: hyperuricemic nephropathy; HUA: hyperuricemia; MDR1: Multi-Drug Resistance Gene-1; PDZK1: PDZ, Domain Protein 1; PO: potassium oxonate; P-gp: P-glycoprotein; SCFAs: Short-chain fatty acids; ZO-1: zonula occludens-1.

### 6.1 Regulation of gut microbiota composition

The intricate equilibrium of gut microbiota composition is strongly intertwined with UA metabolism and several pathophysiological conditions. Natural products showed a promising role in enhancing UA metabolism and alleviating associated secondary disorders through regulating gut microbiota composition, typically exerting an augmentation of beneficial bacteria and a diminution of harmful bacteria (Chen et al., 2023b). Generally, to resist detrimental Bacteroidaceae, bacterial genera such as *Bifidobacterium*, Lactobacillaceae, and Lachnospiraceae, which represent the beneficial bacterial spectrum, usually grow superiorly and can be strengthened by natural products (Amatjan et al., 2023). For instance, studies have documented that rhein can upregulate *Lactobacillus*, thereby enhancing purine metabolism and mitigating secondary manifestations of HUA (Wu et al., 2020; Cao et al., 2023). Moreover, supplementation with Radix Astragali and berberine in HUA model mice could reduce the abundance of Prevotellaceae, Rikenellaceae, and Bacteroidaceae, while augmenting Lactobacillaceae*, Coprococcus, Bacteroides, Akkermansia*, and *Prevotella*, which concurrently assist of regulating XO’ activation and renal function, and safeguarding mice against HUA induced damage (Shan et al., 2022; Deng et al., 2023). In a clinical trial, patients with renal failure were administered inulin-type prebiotics, and results showed that it could elevate the ratio of *Firmicutes* to *Bacteroidetes*, with the majority of bacteria enriched in their fecal matter correlated with UA degradation (He et al., 2022). Numerous studies have consistently demonstrated that an augmentation in SCFAs production is frequently associated with increased abundance of beneficial bacteria involved in UA metabolism, confirming the beneficial effect of SCFAs on UA metabolism (Ni et al., 2021). It has reported that both coffee leaf tea (Zhou et al., 2023) and chlorogenic acid (Zhou et al., 2021) exhibited therapeutic effects on hyperuricemic nephropathy (HN) by improving the abundance of SCFAs-produced bacteria, indicating that SCFAs-produced gut bacteria may serve as pivotal targets of natural products to ameliorate diseases associated with dysmetabolism of UA. In conclusion, numerous evidences suggest that the optimization of gut microbiota composition serves as a pivotal step in the regulation of UA metabolic imbalance by natural products.

### 6.2 Maintenance of gut barrier’ integrity

The physiological gut barrier is comprised of physical structure, microflora, and immunological microenvironment, which regulates various physiological activities, including UA metabolism. The physical barrier consists of an epithelial layer rich in tight junction proteins and a mucus layer composed of abundant mucins, and the immunological microenvironment is constituted by immunological tissues and cells (Sylvestre et al., 2023). Recent studies manifested that high levels of UA could impair the gut’s physical barrier and microenvironment (Lv et al., 2020). Interestingly, a report illustrated that natural products could affect UA metabolism by restoring these gut barriers. Administration of the extract of Dendrobium officinale leaves and herbal formulation Qu-zhuo-tong-bi decoction can repair the intestinal barrier damage caused by HUA, manifested by upregulation of ZO-1 and occludin expression, thereby enhances gut mucosal thickness and facilitates the normal excretion of UA (Wen et al., 2020; Li L. Z. et al., 2023). Upon disrupting the gut barrier, inflammatory mediators can readily disseminate into the systemic circulation and exacerbate simultaneous symptoms induced by HUA. Natural extract tuna meat oligopeptide was considered as a potential inhibitor of the TLR4/MyD88/NF-κB signaling, and a study testified that it could suppress TLR4 signaling cascade and NLRP3 inflammasome subsequently, thereby orchestrating the restoration of the gut barrier integrity (Han et al., 2020). Regarding immunological microenvironment coordinates gut immune barrier repairment, a report has manifested that the extract of Aconitum carmichaelii root not only modulated UA metabolsim but also regulated immune-associated siganling proteins, including TLR4 and NOD1, consequently promoting the transcription of tight junction protein in colitis model mice (Fu et al., 2022). Furthermore, a profound interplay between gut microbiota and gut barrier function was exsisted, research elucidated that augmention of SCFAs-generating microflora strains could significantly fortifies gut barrier integrity, and that could be amplified by curcumin supplementation in HN rats (Xu X. et al., 2021). In summary, numerous natural products exhibit potent reparative effects on the intestinal barrier, thereby facilitating the recovery from HUA.

### 6.3 Modulation of inflammatory response

The inflammatory status of the gut interacts with inflammation related to UA metabolism, while natural products with anti-inflammatory properties can suppress inflammatory signal pathways involved in UA metabolism, potentially reducing the risk of HUA and related diseases (El-Tantawy, 2021). Crucial mediators that regulate the intricate interaction between inflammation and UA metabolism are the NLRP3 inflammasome and the NF-κB signaling molecules. A study has shown that *A. japonicus* oligopeptide significantly inhibited the NF-κB signaling pathway and NLRP3 inflammatory vesicle, thereby alleviating HUA-induced inflammation in HUA model mice (Lee et al., 2022). Moreover, recent studies have elucidated that Tuna meat oligopeptides (TMOP) (Han et al., 2020), *Camellia japonica* bee pollen (Xu Y. et al., 2021), *A. japonicus* (EH-JAP) and *Apostichopus leucoprocta* (EH-LEU) (Wan et al., 2020) commonly exhibited inhibitory effects on the TLR4/MyD88/NF-κB signaling pathway. Individually, TMOP could modulate the UA metabolic pathway by inhibiting the phosphorylation of p65-NF-κB also. Meanwhile, *C. japonica* bee pollen could regulate specific gut transport proteins and gut microflora composition. Besides, both EH-JAP and EH-LEU could reduce the abundance of pathogens in the gut tract while exhibiting antagonistic effects on HUA and its induced renal inflammation. Gut inflammation intricately intertwined with various factors underpinning the UA metabolism process. IL-1β and IL-18 are the most pivotal cytokines among these inflammatory molecules, contributing to several pathological processes (Keenan, 2020). UA was considered as a crucial exogenous ligand for the NLRP3 inflammasome, and it reasonably speculated that HUA intensified the inflammatory response processes (Yue et al., 2021). An observation verified that IL-1β and IL-6 were evildoers of initiating systemic inflammation, while chlorogenic acid inhibited their detrimental action via NLRP3 signaling in HUA model mice (Zhou et al., 2021). In summary, natural products emerge as promising candidates for preventing gut tract inflammation and mitigating disorders associated with UA dysmetabolism.

### 6.4 Regulation of purine metabolism

Purine metabolism comprises *de novo* synthesis, nucleotide degradation, and salvage processes, requiring a series of synthetic and catabolic enzymes such as phosphoribosyltransferase, xanthine dehydrogenase, XO, and uricase. UA is the end product of human purine metabolism and is generated directly from the purine metabolite xanthine (Furuhashi, 2020). XO is the crucial rate-limiting enzyme involved in this metabolic pathway, which is generated from the liver and plays a pivotal role in orchestrating purine metabolism and concerting gut microenvironment (Bortolotti et al., 2021). Recent studies indicated that natural products could target XO as inhibitors and effectively reduce UA production, providing a potential therapeutic strategy for HUA (Sun et al., 2024). In addition, other scholars have revealed an intricate interaction between gut flora and XO activation, which could be modulated sophisticatedly by several inherent factors in the gut microecology system (Liu et al., 2022; Shan et al., 2022). Perturbation of gut flora profoundly affects purine metabolism, particularly the presence of *Lactobacillus*, which is closely correlated with the purine catabolic process. These above findings were testified by the flavonoid extract derived from saffron floral bio-residues, which antagonizes HUA by modulating both XO activity and gut flora composition (Chen et al., 2022). Total flavonoids isolated from *Glycyrrhiza uralensis* and rhein ameliorated the disorder of purine metabolism and reduced UA levels in the feces with dysbacteriosis in colitis mice (Wu et al., 2020; Yue et al., 2021). This definite efficacy of natural compounds to improve purine metabolism. Furthermore, HUA-triggered renal epithelial-mesenchymal transition is linked to altered XO activity, and Qi-Zhu-Xie-Zhuo decoction has the potential to reverse this process (Huijuan et al., 2017). Moreover, the classic herbal prescription Ermiao Wan is usually applied for the therapeutic management of HUA. A report indicate that this formula enhances purine degradation, modulates key metabolites like hypoxanthine, xanthine, and UA, thereby combating HUA (Gu et al., 2023). The above studies indicated that natural products could effectively ameliorate purine metabolism in coordination with microbiota to reduce UA production, which provides a potential therapeutic strategy for HUA-induced disorders.

### 6.5 Modulation of gut transporter activity

In recent years, studies have illustrated several transporters located in the gastrointestinal tract epithelium that have significantly contributed to modulating UA metabolism through the reabsorption or excretion of UA in the gut microenvironment. For instance, GLUT9 primarily exhibits the regulation of UA reabsorption into enterocytes, while ABCG2 is responsible for the excretion of UA from enterocytes into the gut lumen. Interestingly, URAT1 exerts both reabsorption and excretion of UA, corresponding to environmental changes in gut microecology (Dalbeth et al., 2021). Notably, recent findings have indicated that natural products may modulate the activity of UA transporters in the gut tract, thus influencing UA excretion and preventing its accumulation (Zhang M. Q. et al., 2023). Supplementation with the extracts from Stevia in HUA model mice could change UA levels by suppressing GLUT9 and enhancing ABCG2 expression, resulting in reduced UA levels in the gut tract and serum, and mitigated HUA-induced damage (Mehmood et al., 2019). Coffee-leaf tea has also be testified as an effective intervention to lower serum UA levels, prevent HUA and kidney damage, through modulating the activities of GLUT9, OAT3, and ABCG2 in the HN model (Zhou et al., 2023). In addition, an *in vitro* study also showed that Dioscin exerted a dual modulatory effect by inhibiting URAT1 expression level while stimulating the activity of ABCG2 to transport UA concurrently (Zhang et al., 2018). Furthermore, utilizing network pharmacology and molecular docking methods revealed that the natural product isobavachin facilitated the reduction of UA by activating ABCG2-mediated bile acid secretion (Luo et al., 2023). These findings provide novel insights into how natural products regulate UA transporters and their intricate interaction to modulate UA metabolism.

## 7 Conclusion and perspective

Despite the remarkable emergence of natural products as a vital resource for drug development in recent decades, the utilization of natural products in clinical intervention for diseases still needs improvement in quantity and diversity. As the third stage in ingesting food or drugs, the gut tract holds a pivotal role in maintaining fluid homeostasis, metabolizing nutrients, and eliminating waste products. And the stability of the gut microecology is imperative for the efficient absorption and utilization of dietary and medicinal compounds (Lu et al., 2020). UA is closely related to diet and subsequent changes in gut microecology, and is directly or indirectly involved in a variety of pathological injuries (Zeng et al., 2019). With the rapid progression of multi-omics technology fosters the establishment of virtual and physical screening databases grounded in natural products (Wilson et al., 2020), the elucidation of gut physiological and pathological microecological environments (Shalon et al., 2023), and the refinement of mathematical model predictions based on gut microecology’s temporal and spatial dynamics (Geng et al., 2021), these advancements have bolstered confidence in targeting gut microecology to modulate UA metabolism. However, constructing a sophisticated natural product screening platform aimed at gut microecology for medicinal advancements to intervene in UA metabolism poses a feasible yet formidable challenge.

Current investigations into natural products confront several significant hurdles. Natural products constitute a mass of compounds derived from plants, animals, microorganisms, and other sources. Screening for potential natural candidates targeting gut microecology can be arduous, as current methodologies often hinge solely on the product’s active component, thereby restricting its broad development and utilization. Additionally, acquiring a comprehensive understanding of the intricate modes of action and diverse targets of natural products, along with their variable curative potentials across disease types, poses a considerable challenge. Therefore, we espouse a balanced approach that incorporates the pursuit of novel natural products with exploring established natural compounds and their underlying mechanisms. This strategy will facilitate the establishment of a comprehensive network encompassing natural products, individual extracts, complex preparations, and disease mechanisms, along with a comprehensive database serving as a repository of fundamental data to cater to the diverse needs of researchers. In addition, aligned with the complexities inherent in natural product discovery, the precise modulation of gut microecology also encounters significant challenges. Gut microecology represents a dynamic and intricately balanced system characterized by the diverse composition of gut microbiota, where even strains within the same genus perform distinct roles. The precise elucidation of the mechanisms of gut microbiota and the factors that shape gut microecology concurrently poses a formidable challenge, given the intricate interplay of numerous regulatory mechanisms influenced by environmental factors, dietary patterns, multi-systemic diseases, and various pathological aspects. In recognition of this complexity, we advocate for the extensive utilization of multi-omics technology to elucidate the diverse functionalities of the same microbiota in various disease models and the distinct functionalities of different microbiota within a single disease model. Furthermore, we support a comprehensive examination of the spectrum of gut microecological alterations across different pathological stages of a given disease, encompassing alterations in microbiota, gut barriers, inflammatory states, and other contributing factors.

Furthermore, the preponderance of research about UA metabolism primarily focuses on its synthesis and elimination, specifically the activity of XO and the expression of UA transporters. Nonetheless, in the absence of clinical manifestations, patients often ignore HUA. As a result, to augment our comprehension of the dynamic role of UA in pathological processes, it is crucial to conduct research that concurrently examines UA as both a consequence of lesions and an etiological factor.

In conclusion, Delving into potential targets for modulating UA metabolism within the intricate gut microecology utilizing natural products could offer valuable insights into natural product-based drug development, intricate equilibrium maintenance of gut microbiota, and the intricate regulatory mechanisms underlying UA metabolism. Our comprehensive review demonstrates that extracts of natural products and herbal compounds not only have the potential to regulate gut microecology and UA metabolism but can also indirectly improve UA metabolism by modulating the gut microecology. Specifically, they can regulate gut microbiota composition, restore intestinal barrier integrity, alleviate inflammatory responses, modulate purine metabolism, and influence intestinal transporter function, thereby alleviating HUA and related complications. Our review provides conceptual frameworks and a foundation for more comprehensive and precise investigations into natural product-oriented gut microecology interventions in UA metabolism and associated disorders.
